# Characterisation and prognosis of undiagnosed chronic obstructive pulmonary disease patients at their first hospitalisation

**DOI:** 10.1186/1471-2466-15-4

**Published:** 2015-01-17

**Authors:** Eva Balcells, Elena Gimeno-Santos, Jordi de Batlle, Maria Antonia Ramon, Esther Rodríguez, Marta Benet, Eva Farrero, Antoni Ferrer, Stefano Guerra, Jaume Ferrer, Jaume Sauleda, Joan A Barberà, Àlvar Agustí, Robert Rodriguez-Roisin, Joaquim Gea, Josep M Antó, Judith Garcia-Aymerich, the PAC-COPD Study Group

**Affiliations:** Servei de Pneumologia, Hospital del Mar-IMIM, Passeig Marítim 25-29, 08003 Barcelona, Spain; IMIM (Hospital del Mar Medical Research Institute), Doctor Aiguader 88, 08003 Barcelona, Spain; CIBER de Enfermedades Respiratorias (CIBERES), Recinte Hospital Joan March, Carretera Soller km 12, 07110 Bunyola, Spain; Department of Experimental and Health Sciences, Universitat Pompeu Fabra, Doctor Aiguader 88, 08003 Barcelona, Spain; CREAL- Centre for Research in Environmental Epidemiology, Barcelona Biomedical Research Park, Dr. Aiguader, 88, 08003 Barcelona, Catalonia Spain; CIBER Epidemiología y Salud Pública (CIBERESP), Doctor Aiguader 88, 08003 Barcelona, Spain; FCS Blanquerna, Research in Physiotherapy Group (GReFis), Universitat Ramon Llull, Padilla 326, 08025 Barcelona, Spain; Section of Nutrition and Metabolism, International Agency for Research on Cancer (IARC-WHO), Lyon, France; Servei de Pneumologia, Hospital Universitari Vall d’Hebron, Universitat Autònoma de Barcelona, Barcelona, Spain; Departament de Medicina, Universitat Autònoma de Barcelona, Barcelona, Spain; Servei de Pneumologia, Hospital de Bellvitge, Feixa Llarga s/n, 08907L Hospitalet de Llobregat, Spain; IDIBELL (Institut d’Investigació Biomèdica de Bellvitge), Gran Via de L’Hospitalet 199, 08908 Hospitalet de Llobregat, Spain; Arizona Respiratory Center, Tucson, AZ USA; Servei de Pneumologia, Hospital Universitari Son Espases, Carretera de Valldemosa 79, 07010 Palma de Mallorca, Spain; Institut de Investigació Sanitària de Palma (IdISPa), Carretera de Valldemossa 79, Palma, Spain; Thorax Institute, Hospital Clínic, Barcelona, Spain; Institut d’Investigació Biomèdica Agustí Pi I Sunyer (IDIBAPS), Barcelona, Spain; Universitat de Barcelona, Barcelona, Spain; Fundació Investigació Sanitària Illes Balears (FISIB), Palma de Mallorca, Spain

**Keywords:** Pulmonary disease, Chronic obstructive, Hospitalisation, Cohort studies, Epidemiology, Health services

## Abstract

**Background:**

Under-diagnosis of COPD is an important unmet medical need. We investigated the characteristics and prognosis of hospitalised patients with undiagnosed COPD.

**Methods:**

The PAC-COPD cohort included 342 COPD patients hospitalised for the first time for an exacerbation of COPD (2004–2006). Patients were extensively characterised using sociodemographic, clinical and functional variables, and the cohort was followed-up through 2008. We defined “undiagnosed COPD” by the absence of any self-reported respiratory disease and regular use of any pharmacological respiratory treatment.

**Results:**

Undiagnosed COPD was present in 34% of patients. They were younger (mean age 66 *vs.* 68 years, p = 0.03), reported fewer symptoms (mMRC dyspnoea score, 2.1 *vs.* 2.6, p < 0.01), and had a better health status (SGRQ total score, 29 *vs.* 40, p < 0.01), milder airflow limitation (FEV_1_% ref., 59% *vs.* 49%, p < 0.01), and fewer comorbidities (two or more, 40% *vs.* 56%, p < 0.01) when compared with patients with an established COPD diagnosis. Three months after hospital discharge, 16% of the undiagnosed COPD patients had stopped smoking (*vs.* 5%, p = 0.019). During follow-up, annual hospitalisation rates were lower in undiagnosed COPD patients (0.14 *vs*. 0.25, p < 0.01); however, this difference disappeared after adjustment for severity. Mortality was similar in both groups.

**Conclusions:**

Undiagnosed COPD patients have less severe disease and lower risk of re-hospitalisation when compared with hospitalised patients with known COPD.

**Electronic supplementary material:**

The online version of this article (doi:10.1186/1471-2466-15-4) contains supplementary material, which is available to authorized users.

## Background

Chronic obstructive pulmonary disease (COPD) represents a major public health problem, and its mortality and disability burden is expected to rise in the coming decades [1, 2]. Nonetheless, the majority of studies from general population and primary care have detected that a high proportion of individuals fulfilling COPD diagnosis criteria remain undiagnosed [3–9]. Interestingly, it has been reported that a high proportion of undiagnosed patients already suffer from respiratory symptoms [7, 8]. A recent population-based study demonstrated that even newly diagnosed COPD patients with mild airflow limitation exhibit a significant impairment in their health-related quality of life and certain activities of daily living, when compared with individuals without COPD [9]. Therefore, both researchers and practitioners advocate for early detection strategies aimed at reducing COPD burden through proven health-care interventions [10].

There is a lack of specific information regarding COPD under-diagnosis in patients requiring hospitalisation because of an exacerbation of the disease. Two previous studies in a hospital setting highlighted that one-third of patients had never been diagnosed or treated. One of these studies involved patients who went to the emergency room for COPD exacerbation, and the second study was a small retrospective study of patients admitted to the hospital for the first time for a COPD exacerbation [11, 12]. The current study describes the characteristics of COPD patients who were undiagnosed at the time of their first hospital admission because of a COPD exacerbation and their short- and long-term outcomes.

## Methods

### Study design and ethics

This study was a longitudinal observational analysis conducted within the Phenotype and Course of COPD Project (PAC-COPD) [13]. Briefly, the PAC-COPD study included all patients admitted to nine teaching hospitals in Spain between January 2004 and March 2006 for a first-time COPD exacerbation.

**Figure 1:**
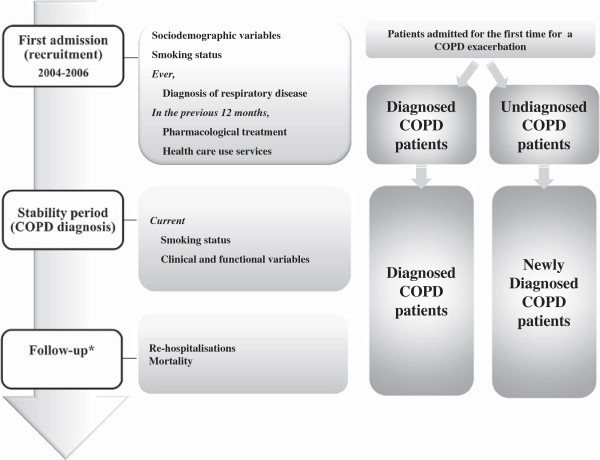
**Design and study population.** *Until Dec 31, 2007 (re-hospitalisations) and Dec 31, 2008 (mortality).

The study design is diagrammed in Figure 1 and included the following features: (i) a recruitment visit (at first hospitalisation due to COPD exacerbation) to obtain sociodemographic variables, smoking status, information about diagnosis and treatment previous to their first hospitalisation, and use of health services during the 12 months preceding their first hospitalisation; (ii) a visit under stable conditions (at least three months after discharge) to collect clinical and functional variables and smoking status; and (iii) a prospective 4-year active follow-up to obtain information about re-hospitalisations and mortality.

During hospitalisation and at discharge, patients received standard information about their disease, smoking cessation advice, as well as pharmacological and non-pharmacological treatment from the attending physician according to local guidelines [14].

The study was approved by the Ethics Committees of all participating hospitals and all patients gave their written informed consent. All patients were actively followed until death or December 31, 2008.

Additional details about the recruitment and follow-up processes have been previously published [13, 15, 16].

### Study population

A diagnosis of COPD was confirmed by spirometry at least three months after discharge when the patient had reached clinical stability. COPD was identified as a post-bronchodilator forced expiratory volume in one second to forced vital capacity ratio (FEV_1_/FVC) of less than 0.7 [17]. At recruitment (first hospitalisation due to COPD exacerbation), patients were asked about their diagnosis with “any respiratory disease” using the following questions: “Are you suffering from any respiratory disease?”, “What is the name of your respiratory disease?”, “When were you diagnosed with this respiratory disease?”, and “Who diagnosed your respiratory disease?”. These questions were previously designed and pilot-tested in COPD patients from the same geographical area [18]. Patients reported any pharmacological treatments they were taking regularly (previous to hospitalisation) for any chronic disease. We defined “undiagnosed COPD” as the absence of any self-reported diagnosis of respiratory disease. In addition, to reduce a potential misclassification due to poor recall, we assumed that patients regularly using any pharmacological respiratory treatment had been previously diagnosed. Once stable conditions were reached and the diagnosis of COPD was confirmed, patients were identified as “newly diagnosed” COPD patients. Details on the exact wording of patients when describing their respiratory disease, time from diagnosis, diagnosing doctor, and respiratory treatment are reported in Additional file 1: Table S1.

For our analysis, disease severity was classified according to FEV_1_ levels as mild, moderate, severe and very severe following the European Respiratory Society and the American Thoracic Society (ERS/ATS) criteria [17].

### Measurements

At recruitment, standardised epidemiological questionnaires were used to collect information on sociodemographic characteristics, smoking status, physical activity (Spanish version of the Yale Physical Activity Survey) [19] and health-care utilisation over the previous 12 months [18]. The Charlson index of comorbidity was obtained from medical records, patient recall and physical examination by an expert pulmonologist [20]. In addition, we obtained the number of visits to a hospital emergency department, primary care emergency department, primary care physician, primary care pulmonologist, and hospital-based pulmonologist over the previous 12 months using standardised epidemiological questionnaires.

When the patient was clinically stable after discharge, the following measurements were obtained: forced spirometry and bronchodilator test, static lung volumes by whole-body plethysmography, diffusing capacity for carbon monoxide (DLco), arterial blood gases analysis while breathing room air at rest, six-minute walking distance (6MWD), body mass index (BMI) and fat-free mass index (FFMI). Patients also answered an epidemiological questionnaire, including a dyspnoea assessment using the mMRC scale, to determine the patient’s smoking status and current pharmacologic treatment information. Health-related quality of life (HRQL) was assessed using the validated Spanish version of St. George’s Respiratory Questionnaire (SGRQ) [21]. Anxiety and depression were evaluated with the Spanish version of the Hospital Anxiety and Depression Scale (HADS) [22, 23].

Detailed information on the methods and sources of the questionnaires and the standardisation of the tests used in the PAC-COPD study has been previously published [13, 16].

### Re-hospitalisations and mortality during follow-up

Information on re-hospitalisations through December 31, 2007 (causes and dates) was obtained for all patients from the Minimum Basic Dataset (CMBD), a national administrative database. According to the 9th revision of the International Classification of Diseases, an admission for COPD exacerbation was defined as any admission with codes 466, 480–486, 490–496, or 518.81 as the main diagnosis. Survival status until December 31, 2008 was obtained from direct interviews with all patients or their relatives. In cases of death, both hospital and primary care registries were checked to verify the exact date.

### Statistical analysis

The sample size was fixed by the primary scientific objectives of the PAC-COPD Study [16]. Before any analysis, we calculated whether the available number of patients (225 patients in the diagnosed group and 117 in the undiagnosed group) would allow for identification of clinically significant differences in outcome between groups (diagnosed *vs*. undiagnosed). Calculations using the GRANMO 5.2 software [24] showed that, accepting an alpha risk of 0.05 in a two-sided test, the statistical power was 84 to recognize as statistically significant the difference in proportion admitted (44% *vs*. 28%, respectively).

Descriptive data are presented as the number and percentage, the mean and standard deviation (SD), or the median and 25th or 75th percentiles, as appropriate. We compared the sociodemographic and clinical variables and use of healthcare resources prior to first hospitalisation according to previous COPD diagnosis status, using Student’s t-test or Mann–Whitney U test for quantitative variables and a Chi squared or Fisher exact test for qualitative variables. We tested the effect of receiving a new COPD diagnosis on quitting smoking by including an interaction term between time (recruitment or stability visit) and diagnosis in a logistic regression model that included smoking and potential confounders (gender, age, the Charlson index of comorbidity, degree of dyspnoea, quality of life, FEV_1_, arterial oxygen tension (PaO_2_)).

Kaplan-Meier curves of time to COPD readmission were plotted according to COPD diagnosis status previous to the baseline admission, and the log-rank test was used to compare differences in readmission-free rates between diagnosed and undiagnosed COPD patients [25]. Because the proportionality assumption held, the association between previous COPD diagnosis and time to COPD readmission was assessed using Cox regression survival-time models [26]. Multivariate models included as covariates all potential confounders that were related to both the exposure and the outcome, or modified the estimates (>10% change in Hazard Ratio) for the remaining variables. Potential covariates included gender, age, marital status, smoking status, quality of life, degree of dyspnoea, BMI, FFMI, the Charlson index of comorbidity, FEV_1_, DLco, Residual Volume/Total Lung Capacity (RV/TLC), PaO_2_, arterial carbon dioxide tension (PaCO_2_), 6MWD, and anxiety and depression. The same approach was to be used to assess the effect of undiagnosis on mortality; however, there were very few deaths during follow-up and this multivariate analysis was not completed. Data analyses were conducted using Stata 10.1 (StataCorp, College Station, TX, USA).

## Results

### Characteristics of patients with undiagnosed COPD

The entire PAC-COPD cohort included 342 patients (93% men) with a mean (SD) age of 67 (9) years and a mean (SD) post-bronchodilator FEV_1_ of 52% (16%) predicted during clinical stability (Table 1). A total of 117 patients (34%) fulfilled the criteria of “undiagnosed COPD”. Table 1 shows the comparisons of sociodemographic and clinical characteristics for these two groups. Undiagnosed patients were younger and more physically active, had fewer symptoms and better health status, and had milder airflow limitation and fewer comorbidities; in addition a higher proportion of these patients reported that they currently smoked (Table 1). A total of 33 (28%) patients with severe COPD and 5 (4%) patients with very severe COPD reported that they had never been diagnosed as having a respiratory disease prior to their first hospitalisation. The Charlson comorbidities are shown in Additional file 1: Table S2.

**Table 1:** **Baseline characteristics of 342 COPD patients recruited at their first hospitalisation for a COPD exacerbation**

	All COPD patients n = 342*	Undiagnosed COPD n = 117 (34%)	Diagnosed COPD n = 225 (66%)	p-value ^†^
Age (years), m (SD)	67 (9)	66 (9)	68 (8)	0.03
Males, n (%)	318 (93)	107 (92)	211(94)	0.43
Married, n (%)	274 (80)	90 (77)	184 (82)	0.29
Less than primary education, n (%)	142 (42)	46 (39)	96 (43)	0.55
Low socioeconomic status (IV-V), n (%)	259 (82)	90 (81)	169 (82)	0.83
Current workers, n (%)	61 (18)	30 (26)	31 (14)	<0.01
Smoking status: current, n (%)	150 (44)	69 (59)	81 (36)	<0.01
Pack-years, m (SD)	69 (40)	67 (38)	70 (41)	0.55
Physical activity (hours/week), m (SD)	33.5 (23.8)	39.5 (23.4)	30.4 (23.5)	0.01
≥2 comorbidities (Charlson index), n (%)	172 (50)	47 (40)	125 (56)	<0.01
Severity of COPD (ERS/ATS), n (%)				
Mild (FEV_1_ ≥ 80%)	19 (5)	14 (12)	5 (2)	<0.01
Moderate (FEV_1_ ≥ 50%, <80%)	164 (48)	65 (56)	99 (44)	
Severe (FEV_1_ ≥ 30%, <50%)	132 (39)	33 (28)	99 (44)	
Very severe (FEV_1_ < 30%)	27 (8)	5 (4)	22 (10)	
FEV_1_ post-bronchodilator (% pred), m (SD)	52 (16)	59 (16)	49 (15)	<0.01
DL_CO_ (% pred.), m (SD)	65 (21)	67 (21)	64 (21)	0.23
RV/TLC (%), m (SD)	56 (10)	52 (10)	58 (9)	<0.01
PaO_2_ (mmHg), m (SD)	74 (11)	75 (10)	74 (11)	0.28
PaCO_2_ (mmHg), m (SD)	41.8 (5.3)	42.2 (5.2)	41.6 (5.4)	0.37
6MWD (m), median (P25-P75)	437 (390–500)	440 (396–502)	437 (373–498)	0.25
Dyspnoea score (mMRC, score 0–4), m (SD)	2.40 (1.06)	2.06 (1.09)	2.59 (0.99)	<0.01
BMI (Kg/m^2^), m (SD)	28.2 (4.7)	28.8 (4.7)	27.9 (4.6)	0.08
FFMI (Kg/m^2^), m (SD)	19.7 (3.1)	19.9 (3.0)	19.5 (3.1)	0.21
SGRQ total score (0 no health impairment to 100 maximum impairment), m (SD)	37 (18)	29 (16)	40 (18)	<0.01
SGRQ symptoms score, m (SD)	48 (18)	45 (16)	50 (18)	<0.01

ERS/ATS: European Respiratory Society/American Thoracic Society; FEV_1_: forced expiratory volume in 1 second; FEV_1_/FVC: forced expiratory volume in 1 second/forced vital capacity; RV/TLC: Residual Volume/Total Lung Capacity; DL_CO_: diffusing capacity for carbon monoxide; PaO_2_: arterial oxygen tension; PaCO_2_: arterial carbon dioxide tension; 6MWD: six-minute walking distance; mMRC: modified Medical Research Council; BMI: body mass index; FFMI: fat-free mass index; SGRQ: St. George’s Respiratory Questionnaire. *Some variables had missing values: 25 in socioeconomic status, one in physical activity, four in dyspnoea, 27 in RV/TLC, 46 in DL_CO_, 11 in PaO_2_, 10 in PaCO_2_, 33 in 6MWD, 13 in FFMI, and four in SGRQ score. ^†^Comparison between undiagnosed and previously diagnosed COPD.

Undiagnosed patients reported a significantly lower use of health care resources due to respiratory symptoms in the 12 months prior to their first hospitalisation for a COPD exacerbation. The number of unscheduled visits to the primary care surgery was similar in both groups (Table 2).

**Table 2:** **Self-reported diagnosis, respiratory treatment and use of health care resources due to respiratory symptoms of 342 COPD patients in the 12 months prior to their first hospitalisation for a COPD exacerbation**

	All COPD patients n = 342	Undiagnosed COPD n = 117 (34%)	Diagnosed COPD n = 225 (66%)	p-value ^†^
	n (%)	n (%)	n (%)	
**COPD diagnosis and treatment**				
COPD diagnosis*	157 (46)	--	157 (70)	--
COPD treatment*	193 (56)	--	193 (86)	--
**Use of health care resources due to respiratory symptoms in the 12 months prior to first COPD hospitalisation**				
At least one visit to hospital emergency department	34 (10)	3 (3)	31 (14)	<0.01
At least one unscheduled visit to primary care	64 (19)	21 (18)	43 (19)	0.79
≥3 visits to any physician	104 (31)	15 (13)	89 (40)	<0.01
≥3 visits to primary care physician	56 (16)	6 (5)	50 (22)	<0.01
≥3 visits to primary care-based pulmonologist	18 (5)	1 (1)	17 (8)	<0.01
≥3 visits to hospital-based pulmonologist	2 (1)	0 (0)	2 (1)	0.55

*See Additional file 1: Table S1 in for details.

^†^Comparison between undiagnosed and diagnosed COPD.

### Short-term effects associated with a COPD diagnosis

**Figure 2:**
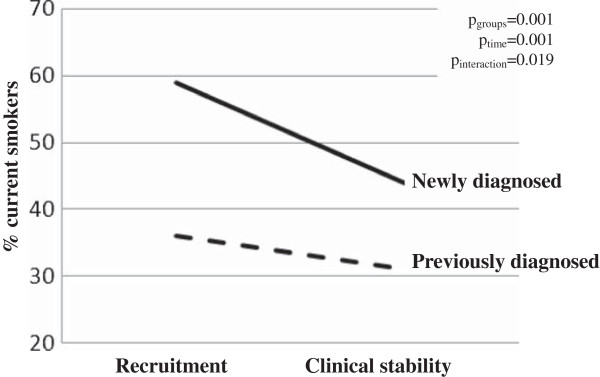
**Short-term effects of a new COPD diagnosis on smoking cessation.** P-values were obtained from a logistic regression model with active smoking as the outcome and the interaction between diagnosis status and time (period) included as explanatory variables. For further explanations, see the main manuscript text.

Figure 2 shows the short-term effects associated with a COPD diagnosis on smoking cessation. The proportion of current smokers after hospital discharge decreased significantly more in newly diagnosed COPD patients than in those with a previous COPD diagnosis (16% *vs.* 5%). Despite significantly different baseline values at hospitalisation (Figure 2), the interaction between diagnosis group and time was significant (p = 0.019).

### Long-term prognosis of newly diagnosed COPD patients

**Figure 3:**
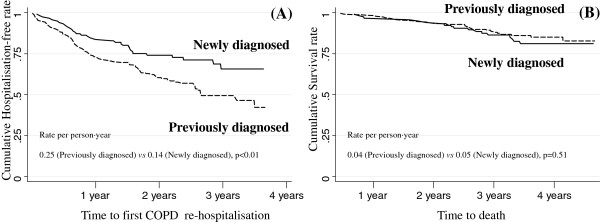
**Kaplan-Meier curves show the cumulative hospitalisation-free rate (panel A) and survival rate (panel B) according to previous COPD diagnosis.**

During a mean (SD) of 1.87 (0.98) years of follow-up, 44% of previously diagnosed patients and 28% of newly diagnosed required re-hospitalisation. This corresponds to 0.25 and 0.14 annual hospitalisation rates (p < 0.01), respectively (Figure 3, panel A). However, this risk of re-hospitalisation was similar in both groups after adjusting for other covariates in a Cox regression multivariate model (Table 3). The proportion of patients who required admission was higher in previously diagnosed patients when compared with newly diagnosed patients for the mild, moderate and severe spirometric COPD groups (20% vs. 7%, 36% vs. 23% and 49% vs. 36%, respectively). The proportion of patients within the very severe COPD group who required admission was 63% in previously diagnosed patients and 100% for newly diagnosed patients; however, the very small sample size prevented any statistical comparisons.

During a mean (SD) of 3.28 (0.85) years, overall survival rates (Figure 3, panel B) of previously diagnosed and newly diagnosed patients were similar (87% and 84%, respectively; p = 0.51) at all severity stages (80% and 93% in mild, 92% and 85% in moderate, 87% and 81% in severe, and 64% and 60% in very severe patients).

**Table 3:** **Association between previous COPD diagnosis and subsequent COPD hospitalisations**

	Crude model		Adjusted model*	
	HR ***(95% CI)***	p-value	HR ***(95% CI)***	p-value
**Previously diagnosed COPD**	1.00		1.00	
**Newly diagnosed COPD**	0.564 (0.380-0.836)	<0.01	0.858 (0.551-1.338)	0.50
**Dyspnoea score (mMRC, score 0–4)**	--	--	1.234 (1.005-1.515)	0.04
**BMI (Kg/m** ^**2**^ **)**	--	--	0.961 (0.919-1.005)	0.08
**RV/TLC (%)**	--	--	1.025 (1.000-1.050)	0.04
**FEV**_**1**_ **post-bronchodilator (% pred)**	--	--	0.994 (0.977-1.011)	0.51

HR: hazard ratio; CI: confidence interval; mMRC: modified Medical Research Council; BMI: body mass index; RV/TLC: Residual Volume/Total Lung Capacity; FEV_1_: forced expiratory volume in 1 second.

*Final models were adjusted to account for negative confounding, i.e., that the apparently protective effect of undiagnosed COPD is due to a lower clinical severity of the disease. Other potential confounders (see text) were tested but not included because they were not independently related to both the exposure and the outcome, nor did these confounders modify (>10% change in Hazard Ratio) the estimates for the remaining variables.

## Discussion

This study has three main findings: *(1)* undiagnosed patients (34% of all patients hospitalised for the first time because of an exacerbation of COPD) have milder airflow limitation, fewer symptoms, fewer comorbidities, and better HRQL when compared with patients with a previous diagnosis of COPD; *(2)* establishing a COPD diagnosis is associated with a positive short-term effect on smoking cessation; and *(3)* undiagnosed patients have a lower risk of re-hospitalisations but a similar mortality after hospitalisation when adjusted for severity of illness and covariates.

A high prevalence of COPD under-diagnosis has been frequently reported, both in population based-studies and in primary care settings [3–9]. In contrast, there is little information available regarding COPD under-diagnosis in hospitalised patients. Our study confirms that undiagnosed COPD is not confined to the general population or primary care. We determined that one-third of patients admitted for the first time for a COPD exacerbation were undiagnosed. This finding is in accordance with a previous Italian study of patients attending the emergency room because of a COPD exacerbationand a retrospective study of patients admitted in a UK hospital for the first time for a COPD exacerbation [11, 12]. Importantly, the hospital-based design and the thorough characterisation of the patients in our study prevented the inclusion of healthy subjects with age-related airflow limitation.

The substantial differences observed between diagnosed and undiagnosed patients deserve special consideration. In our cohort, undiagnosed patients were younger, had less severe airflow limitation and a better HRQL. These findings confirm several previous population-based studies with similar observations [8, 9, 27]. In contrast, Zoia *et al.* did not find differences in age and severity based on previous COPD diagnosis in the hospital setting [11]; however, their diagnosed patients had more comorbidities when compared with undiagnosed patients [11]. It is possible that the lack of diagnosis (hence, treatment) may have resulted in an “earlier” first hospital admission for a COPD exacerbation, when the patient still had mild-to-moderate COPD [15]. In fact, our findings indicated that undiagnosed COPD may be related to a lack of primary care interventions prior to the first admission (Table 3). Unfortunately, specific information about these interventions, such as smoking cessation advice, was not recorded in the PAC-COPD study.

Similar to the report by Zoia *et al.*, we identified a higher proportion of current smokers in the undiagnosed group when compared with the diagnosed group [11]. We also observed that the establishment of a COPD diagnosis was associated with a significant reduction in current smokers (Figure 2). This finding is similar to previous reports that showed that smokers with airflow limitation had significantly higher smoking cessation rates than those with normal spirometry [28, 29]. These data identify a potentially important window of opportunity for therapeutic intervention.

The re-hospitalisation rate was lower in newly diagnosed COPD patients following their first admission (Figure 3, panel A); however, this decreased risk was not significant after multivariable adjustments (Table 3), indicating that the protective effect of undiagnosed COPD was likely due to a lower severity of the disease. This interpretation is challenged by the lack of differences in mortality during follow-up (Figure 3, panel B), and a better prognosis is expected in undiagnosed patients with a milder disease. Thus, this observation requires further research. One potential explanation is that cardiovascular disease might play a more relevant role in undiagnosed patients because the majority were active smokers and had milder COPD. This idea is supported by previous studies that consistently showed the causes of death in patients with mild COPD were predominantly cancer and cardiovascular disease, while deaths due to respiratory disease became more common with increasing COPD severity [30]. In our study, there were very few deaths during follow-up. Therefore, the sample size was too small to analyse differences in cause of death between groups.

Clinical features and outcomes of newly diagnosed COPD patients highlighted the clinical relevance of pursuing a correct diagnosis in all hospitalised patients and applying the appropriate corresponding health measures. A recent report by Suissa *et al.* [31] identified two strategic targets for the management of COPD patients during their first hospitalisation. First, the second hospitalisation should be delayed as much as possible because subsequent exacerbations increase exponentially in frequency and intensity. Second, improved treatment is needed to reduce early mortality [31].

Some limitations of our study should be addressed. Firstly, self-reported information about COPD diagnosis rather than objective medical records could lead to misclassification. Secondly, the very small number of undiagnosed patients with very severe COPD has limited our analysis with regard to this specific subgroup. Finally, our results regarding the extent of COPD under-diagnosis and the clinical profile of these patients may not be able to be generalised to other health care systems; however, the effect of the lack of COPD diagnosis on subsequent hospitalisations and mortality are likely to be generally applicable.

The strengths of our study included the large cohort of COPD patients, and their homogeneity with respect to incipient COPD hospitalisations, the wide spectrum of disease severity, and length of follow up. Furthermore, the comprehensive multidimensional assessment used in our study allowed adjustments for potential confounders.

## Conclusions

This study showed that approximately one-third of patients hospitalised for the first time because of a COPD exacerbation had not been previously diagnosed (hence, treated). In addition, patients generally exhibited less severe disease, and their risk of re-hospitalisation was lower when compared with patients who were hospitalised with an established COPD diagnosis. First admission due to COPD exacerbation provides a window of opportunity for early treatment, in particular for smoking cessation intervention.

## Authors’ information

The “Phenotype and Course of COPD (PAC-COPD)” Study Group: Centre for Research in Environmental Epidemiology (CREAL), Barcelona: Josep M Antó (Principal Investigator), Judith Garcia-Aymerich (project coordinator), Marta Benet, Jordi de Batlle, Ignasi Serra, David Donaire-Gonzalez, Stefano Guerra; Hospital del Mar-IMIM, Barcelona: Joaquim Gea (centre coordinator), Eva Balcells, Àngel Gayete, Mauricio Orozco-Levi, Ivan Vollmer; Hospital Clínic-Institut D’Investigacions Biomèdiques August Pi i Sunyer (IDIBAPS), Barcelona: Joan Albert Barberà (centre coordinator), Federico P Gómez, Carles Paré, Josep Roca, Robert Rodriguez-Roisin, Xavier Freixa, Diego A Rodriguez, Elena Gimeno-Santos, Karina Portillo; Hospital General Universitari Vall D’Hebron, Barcelona: Jaume Ferrer (centre coordinator), Jordi Andreu, Esther Pallissa, Esther Rodríguez; Hospital de la Santa Creu i Sant Pau, Barcelona: Pere Casan (centre coordinator), Rosa Güell, Ana Giménez; Hospital Universitari Germans Trias i Pujol, Badalona: Eduard Monsó (centre coordinator), Alicia Marín, Josep Morera; Hospital Universitari de Bellvitge, L’Hospitalet de Llobregat: Eva Farrero (centre coordinator), Joan Escarrabill; Hospital de Sabadell, Corporació Parc Taulí, Institut Universitari Parc Taulí (Universitat Autònoma de Barcelona), Sabadell: Antoni Ferrer (centre coordinator); Hospital Universitari Son Dureta, Palma de Mallorca: Jaume Sauleda (centre coordinator), Àlvar G Agustí, Bernat Togores; Hospital de Cruces, Barakaldo: Juan Bautista Gáldiz (centre coordinator), Lorena López; Hospital General Universitari, València: José Belda.

## Electronic supplementary material

Additional file 1: Table S1: Characteristics of respiratory diagnoses and pharmacological treatments prior to the first admission for COPD exacerbation in diagnosed COPD patients (n = 225). **Table S2.** Charlson comorbidities in 342 COPD patients recruited at their first hospitalisation for a COPD exacerbation. Comparison between undiagnosed and previously diagnosed COPD patients. (DOCX 33 KB)

## Abbreviations

COPD: Chronic obstructive pulmonary disease
FEV_1_/FVC: Post-bronchodilator forced expiratory volume in one second to forced vital capacity ratio
FEV_1_: Post-bronchodilator forced expiratory volume in one second
ERS/ATS: European Respiratory Society/American Thoracic Society
GOLD: Global initiative for chronic obstructive lung disease
mMRC: Modified medical research council
DLco: Diffusing capacity for carbon monoxide
6MWD: Six-minute walking distance
BMI: Body mass index
FFMI: Fat-free mass index
HRQL: Health-related quality of life
SGRQ: St. George’s respiratory Questionnaire
HADS: Hospital anxiety and depression scale
CMBD: Minimum Basic Dataset
SD: Standard deviation
RV/TLC: Residual volume/total lung capacity
PaO_2_: Arterial oxygen tension
PaCO_2_: Arterial carbon dioxide tension.
