# Supplementary material for: Characterisation and prognosis of undiagnosed chronic obstructive pulmonary disease patients at their first hospitalisation
Source: BMC Pulm Med. 2015 Jan 17;15:4. doi: 10.1186/1471-2466-15-4 (PMC4360934; doi:10.1186/1471-2466-15-4)
Supplement: Supplementary file 1 — Additional file 1: Table S1: Characteristics of respiratory diagnoses and pharmacological treatments prior to the first admission for COPD exacerbation in diagnosed COPD patients (n = 225). Table S2. Charlson comorbidities in 342 COPD patients recruited at their first hospitalisation for a COPD exacerbation. Comparison between undiagnosed and previously diagnosed COPD patients. (DOCX 33 KB) [file 12890_2015_646_MOESM1_ESM.docx]

# Additional file 1

**Table S1. Characteristics of respiratory diagnoses and pharmacological treatments prior to the first admission for COPD exacerbation in diagnosed COPD patients (n=225)**

| **Self-reported diagnosis***, n (%)  Bronchi-related respiratory disease  Chronic bronchitis  Respiratory symptoms  “Unknown name of the respiratory disease”  COPD  Emphysema  Asthma/asthmatic bronchitis  Acute bronchitis/pneumonia  Tobacco-related respiratory disease  Respiratory failure | n=157 | 63 (40)  34 (22)  14 (9)  13 (8)  10 (6)  8 (5)  7 (5)  4 (3)  2 (1)  2 (1) |
| --- | --- | --- |
| **Time since diagnosis (years), median (P25-P75)** | n=148 | 8.5 (3-16) |
| **Physician who diagnosed respiratory disease, n (%)**  Primary care physician  Primary care pulmonologist  Hospital-based pulmonologist  Private physician  Other speciality physician or health care professional  Other person | n=157 | 68 (43)  43 (27)  30 (19)  6 (4)  4 (3)  6 (4) |
| **Treatment** *(more than one allowed)*  Short-acting ß_2-_agonists (SABA), n (%)  Long-acting ß_2-_agonists (LABA), n (%)  Short-acting anticholinergics, n (%)  Long-acting anticholinergics, n (%)  Methylxantines, n (%)  Inhaled corticosteroids (ICS,) n (%)  Oral corticosteroids, n (%)  Antioxidants, n (%)  Mucolytics, n (%)  Combination LABA and tiotropium, n (%)  Combination LABA and ipratropium, n (%)  Combination LABA and ICS, n (%)  Combination LABA, ICS and tiotropium, n (%)  Combination LABA, ICS, tiotropium and teophyline, n (%) | n=193 | 99 (51)  124 (64)  43 (22)  85 (44)  15 (8)  122 (63)  4 (2)  5 (3)  5 (3)  73 (38)  18 (9)  106 (55)  58 (30)  7 (4) |

^*^ According to specific wording used by patients, ordered by frequency.

**Table S2. Charlson comorbidities in 342 COPD patients recruited at their first hospitalisation for a COPD exacerbation. Comparison between undiagnosed and previously diagnosed COPD patients.**

|  | **All COPD patients**  **n=342** | **Undiagnosed COPD**  **n=117 (34%)** | **Diagnosed**  **COPD**  **n=225 (66%)** | **p-value** |
| --- | --- | --- | --- | --- |
| Myocardial infarction, n (%) | 34 (9.9) | 5 (4.3) | 29 (12.9) | 0.012 |
| Congestive heart failure, n (%) | 17 (5.0) | 5 (4.3) | 12 (5.3) | 0.669 |
| Peripheral vascular disease, n (%) | 32 (9.4) | 11 (9.4) | 21(9.3) | 0.984 |
| Cerebrovascular disease, n (%) | 12 (3.5) | 3 (2.5) | 9 (4.0) | 0.758 |
| Connective tissue disease, n (%) | 6 (1.8) | 2 (1.7) | 4 (1.8) | 0.964 |
| Peptic ulcer disease, n (%) | 37 (10.8) | 8 (6.8) | 29 (12.9) | 0.087 |
| Mild liver disease, n (%) | 14 (4.1) | 4 (3.4) | 10 (4.4) | 0.779 |
| Moderate or severe liver disease, n (%) | 2 (0.6) | 1 (0.9) | 1 (0.4) | 0.637 |
| Hemiplegia, n (%) | 2 (0.6) | 2 (1.7) | - | 0.116 |
| Diabetes, n (%) | 61 (17.8) | 17 (14.5) | 44 (19.6) | 0.249 |
| Diabetes with end organ damage, n (%) | 6 (1.8) | 2 (1.7) | 4 (1.8) | 0.964 |
| Any malignancy, n (%) | 33 (9.6) | 12 (10.3) | 21 (9.3) | 0.784 |
| Moderate or severe renal disease, n (%) | 20 (5.8) | 6 (5.1) | 14 (6.2) | 0.683 |
| ≥2 comorbidities (Charlson index), n (%) | 172 (50) | 47 (40) | 125 (56) | < 0.01 |

Other comorbidities included in the Charlson index (e.g., dementia, metastatic malignancies or AIDS) are not shown because of the lack of patients with such diseases.

# 
